# The gene structure and hypervariability of the complete *Penaeus monodon* Dscam gene

**DOI:** 10.1038/s41598-019-52656-x

**Published:** 2019-11-12

**Authors:** Kantamas Apitanyasai, Shiao-Wei Huang, Tze Hann Ng, Shu-Ting He, Yu-Hsun Huang, Shen-Po Chiu, Kuan-Chien Tseng, Shih-Shun Lin, Wen-Chi Chang, James G. Baldwin-Brown, Anthony D. Long, Chu-Fang Lo, Hon-Tsen Yu, Han-Ching Wang

**Affiliations:** 1 Department of Biotechnology and Bioindustry Sciences, National Cheng Kung University, Tainan, Taiwan; 2 International Center for the Scientific Development of Shrimp Aquaculture, National Cheng Kung University, Tainan, Taiwan; 3 Department of Life Sciences, National Taiwan University, Taipei, Taiwan; 4 Department of Life Sciences, National Cheng Kung University, Tainan, Taiwan; 5 Institute of Biotechnology, National Taiwan University, Taipei, Taiwan; 6 Institute of Tropical Plant Sciences, National Cheng Kung University, Tainan, Taiwan; 7 Department of Ecology and Evolutionary Biology, University of California, Irvine, Irvine, California USA

**Keywords:** Molecular biology, Transcriptomics, Open reading frames

## Abstract

Using two advanced sequencing approaches, Illumina and PacBio, we derive the entire Dscam gene from an M2 assembly of the complete *Penaeus monodon* genome. The *P. monodon* Dscam (*Pm*Dscam) gene is ~266 kbp, with a total of 44 exons, 5 of which are subject to alternative splicing. *Pm*Dscam has a conserved architectural structure consisting of an extracellular region with hypervariable Ig domains, a transmembrane domain, and a cytoplasmic tail. We show that, contrary to a previous report, there are in fact 26, 81 and 26 alternative exons in N-terminal Ig2, N-terminal Ig3 and the entirety of Ig7, respectively. We also identified two alternatively spliced exons in the cytoplasmic tail, with transmembrane domains in exon variants 32.1 and 32.2, and stop codons in exon variants 44.1 and 44.2. This means that alternative splicing is involved in the selection of the stop codon. There are also 7 non-constitutive cytoplasmic tail exons that can either be included or skipped. Alternative splicing and the non-constitutive exons together produce more than 21 million isoform combinations from one *Pm*Dscam locus in the *P. monodon* gene. A public-facing database that allows BLAST searches of all 175 exons in the *Pm*Dscam gene has been established at http://pmdscam.dbbs.ncku.edu.tw/.

## Introduction

Dscam belongs to the immunoglobulin (Ig) superfamily gene, and it was first identified in the human chromosome in relation to the development of neuronal connectivity^1^. This gene also plays several important roles in the development of the nervous system in insects^2–4^. Structurally, Dscam consists of 10 Ig domains and six fibronectin type III repeats connected to a transmembrane domain and a cytoplasmic tail^5^. The Dscam gene is hypervariable, with three large tandem arrays located on the N-terminal of Ig2, the N-terminal of Ig3 and the entire Ig7 domain, with each array having many near-duplicate exons^3,5–7^. In *Drosophila*, this allows thousands of Dscam isoforms to be generated through mutually exclusive alternative splicing of the near-duplicate exons^8,9^. The resulting isoforms act as axon guidance receptors in the nervous system and also, at least in insects such as the mosquito, as immune receptors that are capable of recognizing diverse pathogens^2,3,5^. In some arthropods, Dscam plays an essential role in immunity by recognizing specific pathogens, and producing pathogen-specific isoforms in response to immune challenge^3,10–15^. Dscam is also potentially able to generate a specific, long-lasting immune response, and with its hypervariability, it has been hypothesized to be an ortholog of antibody genes in vertebrates^16,17^. Functionally, Dscam provides arthropods with an “immunological memory” and supports a novel immune mechanism (“innate immunity with specificity” or “immune priming”) which allows the innate immune system to exhibit characteristics of adaptive immunity^18–20^.

Dscam protein forms a horse-shoe shaped structure comprised of the first four extracellular Ig domains, with two surface epitopes, epitope I and epitope II, formed by part of the Ig2 and Ig3 domains. Epitope I is involved in homophilic binding specificity, whereas epitope II is hypothesized to be involved in pathogen recognition^21,22^. Originally, Dscam was thought to occur only as a membrane-bound form with a transmembrane domain (TM) and a cytoplasmic tail, and although Dscam can be secreted from cells in *Drosophila*, this can only be achieved by proteolytic activity. Surprisingly, however, it was subsequently found that both shrimp (*Litopenaeus vannamei* and *Penaeus monodon*) and crab (*Eriocheir sinensis*) express a unique tail-less form of Dscam that had neither a transmembrane domain nor cytoplasmic tail^10,16,23,24^. Type III polyadenylation was thought to provide a mechanism that would generate both membrane-bound Dscam and tail-less Dscam^16^.

In the present study, to expand upon and correct our previous understanding of shrimp Dscam, we used hybrid assembly and two advanced sequencing approaches, Illumina and PacBio, to construct an M2 assembly of the entire *P. monodon* genome, from which we derive a draft of the Dscam gene. We show that in *Penaeus monodon* Dscam (*Pm*Dscam), the exons in Ig2, Ig3 and Ig7 are in fact derived from 26, 81 and 26 mutually exclusive alternative variants, respectively. Based on our new transcriptomics data, we were also able to show that *Pm*Dscam has a relatively complex cytoplasmic tail structure that is distinct from insect Dscam. Several highly conserved functional motifs were discovered in the cytoplasmic tail. In addition to our structural analysis of the *Pm*Dscam gene, we also found that most of the alternative exons in the gene were selected in both nervous and immune-related cells. We also show that the various alternatively spliced exons in the extracellular region together with the alternatively spliced and non-constitutive exons in the cytoplasmic tail are capable of generating over 21 million distinct protein isoforms.

## Results

### Construction of the *Pm*Dscam gene from the sequencing and M2 assembly of the whole *P. monodon* genome

**Figure 1:**
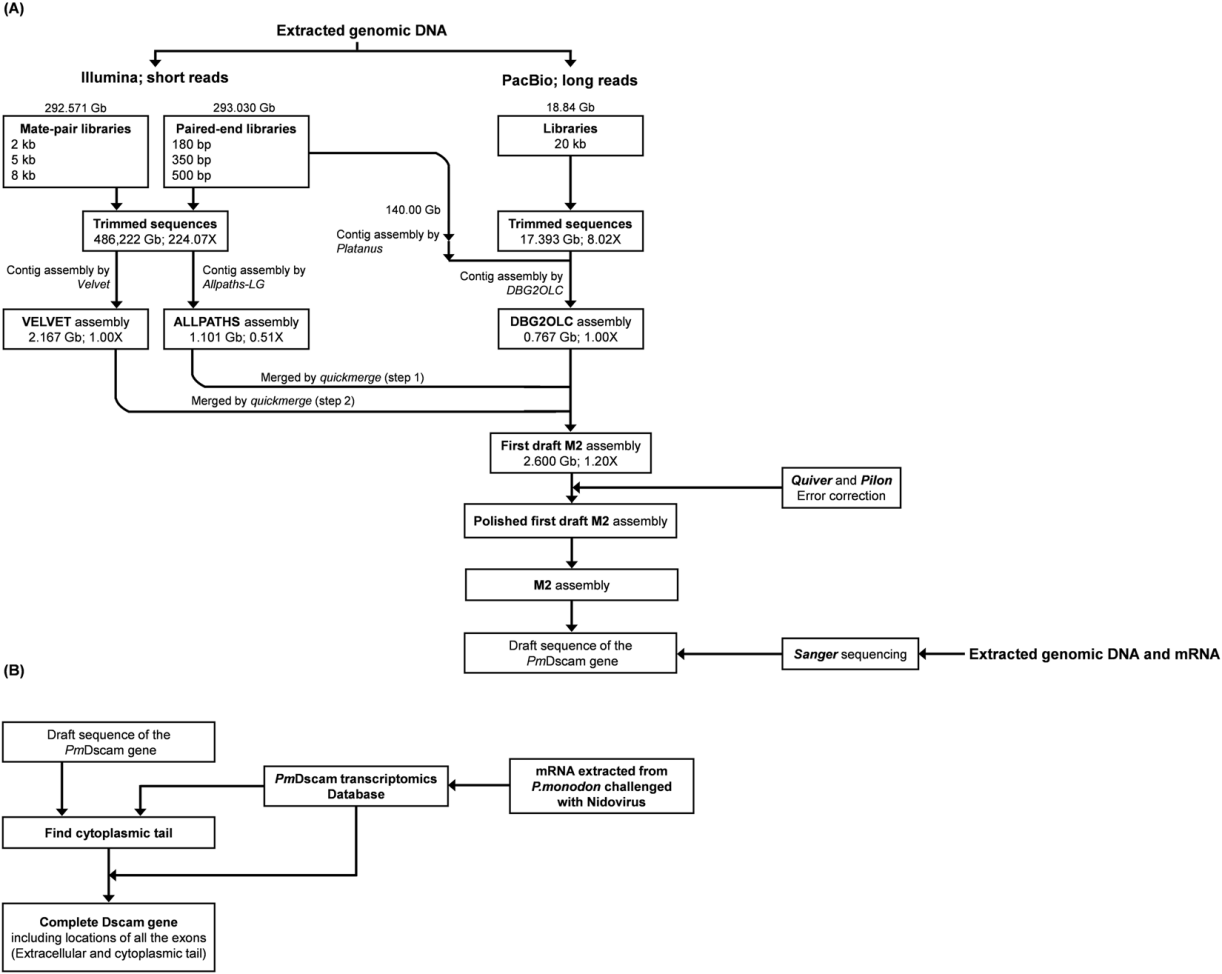
Strategies and genomic sequencing methods. (**A**) Construction and characterization of the polished M2 assembly of the complete *P. monodon* genome that was used to produce a draft sequence of the *Pm*Dscam Dscam gene. (**B**) Analysis steps used to determine the *Pm*Dscam cytoplasmic tail and location of the cytoplasmic tail exons.

**Figure 2:**
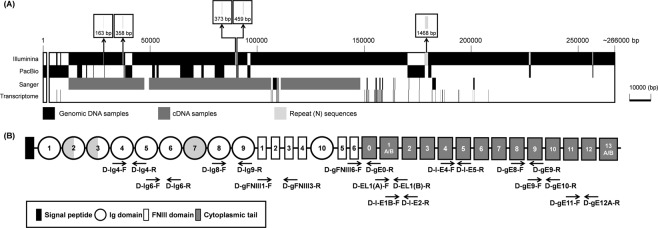
Schematic diagram of the *Penaeus monodon* Dscam gene structure. (**A**) *Pm*Dscam genomic DNA spans 266 kbp. PacBio and Illumina sequencing were used to characterize the entire gene, with Sanger sequencing used to fill some gaps and confirm sequences. Transcriptomics data were used to identify the cytoplasmic tail and some parts of the extracellular region. Samples used for sequencing were derived from both genomic DNA (black) and cDNA (dark grey) samples. The gap near the N-terminal corresponds to a part of the 5′-UTR that we were unable to find in the gene. The boxes above the schematic show the location of five other parts of the gene that contain unknown repeat sequences (N). (**B**) Location of primers for PCR amplification and Sanger sequencing of the *Pm*Dscam gene. Samples were extracted from both genomic DNA and cDNA.

The procedures illustrated in Fig. 1 produced a first draft M2 assembly which had the highest contiguity of any assembly that we generated, with an N50 of 5.1 kb in 2.2 million contigs. The final assembly size was 2.6 Gb (Table S1; Fig. S1). After a draft PmDscam gene was derived from the polished M2 assembly, most of the remaining gaps in the *Pm*Dscam sequence were closed by PCR amplification Sanger sequencing (Fig. 1A). The cytoplasmic tail was determined as shown in Fig. 1B, and the complete *Penaeus monodon* Dscam gene was found to have a size of approximately 266 kbp (Fig. 2). Figure 2 also shows how the three platforms and the transcriptomics data were used to build this construction.

### *Penaeus monodon* Dscam gene organization

**Figure 3:**
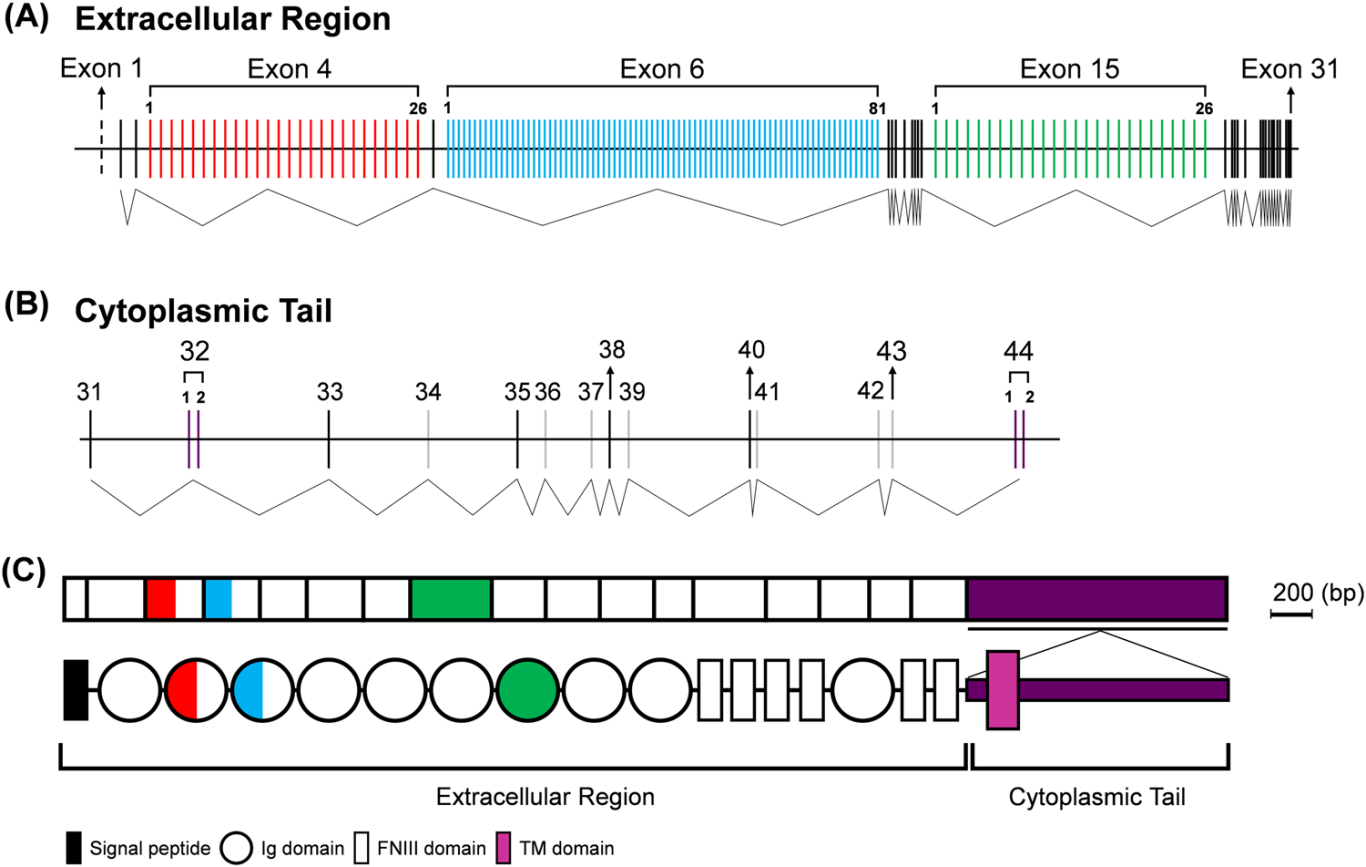
Organization of the *Pm*Dscam gene. The *Pm*Dscam gene consists of 175 exons and/or exon variants: 31 exons are constitutive (black lines), 7 exons (all in the cytoplasmic tail) can be either inserted or skipped (gray lines), and 137 exon variants are subject to mutually exclusive alternative splicing (colored lines). (**A**) The *Pm*Dscam extracellular region is encoded by exon 1 to exon 31. The variable regions are exon 4 (red), exon 6 (blue) and exon 15 (green), which contain 26 variants, 81 variants and 26 variants, respectively. The dashed line represents exon 1, which was not identified from the *P. monodon* gene. (**B**) The cytoplasmic tail is encoded by exon 31 to exon 44. The variable regions are exon 32 and exon 44, with each of these two exons derived from two mutually exclusive variants; that is, after RNA splicing, each transcript contains one of the alternative variants for each of these exons. (**C**) The extracellular region of *Pm*Dscam mRNA (upper panel) contains both constitutive exons (white) and exons that are subject to mutually exclusive alternative splicing. Alternatively spliced exons encode the N-terminal half of Ig2 (red), the N-terminal half of Ig3 (blue), and the entirety of Ig7 (green). In the cytoplasmic tail (purple), both the transmembrane domain (exon 32) and exon 44 are subject to mutually exclusive alternative splicing. The *Pm*Dscam protein structure (lower panel) is comprised of the extracellular region, which contains 10 immunoglobulin (Ig) domains and six fibronectin type 3 (FNIII) domains, followed by the cytoplasmic tail.

While our previous study of *Pm*Dscam was based only on cDNA transcripts^16^, here the assembled *P. monodon Pm*Dscam gene reveals for the first time the entire gene structure. The *Pm*Dscam gene contains a total of 44 exons (Fig. 3), with 137 exon variants that are subject to mutually exclusive alternative splicing. Unfortunately, however, even with the PacBio data, Sanger sequencing, and the cDNA trancsripts, we were unable to identify the 5′-UTR of Dscam that is presumably located in exon 1. This 5′-UTR has been identified in other crustacean species^4,15^, and it remains unclear why it could not be found in *Pm*Dscam. The *Pm*Dscam gene is organized into two main parts: the extracellular region (Fig. 3A) and the cytoplasmic tail (Fig. 3B). The extracellular region of *Pm*Dscam has three alternatively spliced exons, with exons 4, 6 and 15 being derived from the mutually exclusive splicing of 26, 81 and 26 variants, respectively (Fig. 3A). Meanwhile, the cytoplasmic tail has two alternatively spliced exons, exon 32 and exon 44, both of which are derived from two mutually exclusive variants (Fig. 3B). The mature mRNA thus consists of a protein with the same conserved structure that is seen in other arthropods^25^, i.e. a protein that includes immunoglobulin (Ig) domains, fibronectin type III repeats (FNIII) and a transmembrane domain (TM) in the configuration 9(Ig)-4(FNIII)-Ig-2(FNIII)-TM-cytoplasmic tail (Fig. 3C, lower panel).

### Analysis of *Pm*Dscam hypervariable regions

**Figure 4:**
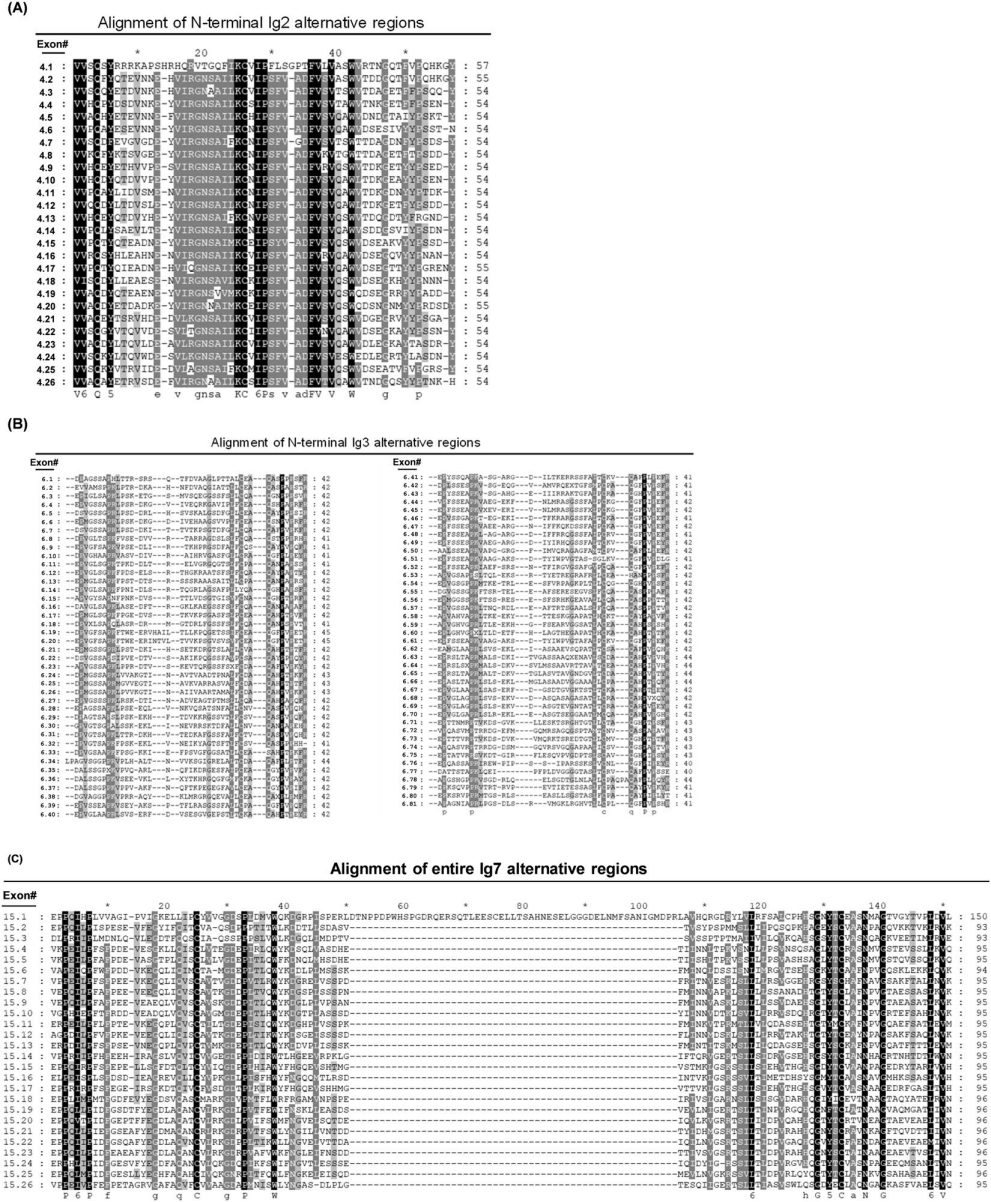
Multiple amino acid sequence alignments of each of the *Pm*Dscam extracellular variable regions. (**A**) 26 variants encode the N-terminal Ig2 domain in the Ig2 exon 4 cluster. (**B**) 81 variants encode the N-terminal Ig3 domain in the Ig3 exon 6 cluster. (**C**) 26 variants encode the entire Ig7 domain in the Ig7 exon 15 cluster. The total number of amino acids for each isoform is indicated on the right. Identical (black) and similar (grey and light grey) amino acids are indicated. Exon#: the exon numbers correspond to the exon’s location in the *Pm*Dscam gene.

First, to identify the hypervariable regions of Ig2, Ig3 and Ig7 in the *Pm*Dscam gene, we searched for the conserved amino acid sequences of isoform variants from each domain. Once identified, the multiple hypervariable exons variants were checked manually and a total of 26, 81 and 26 spliced forms of the exons variants encoding Ig2, Ig3 and Ig7 were detected, respectively. These numbers are in contrast to those in Chou *et al*.^16^, where the number of exon variants in Ig2, Ig3 and Ig7 were reported to be 28, 43 and 19, respectively, from cDNA cloning. The isoform sequences from each domain were aligned using Clustal Omega and Genedoc software, and the resulting amino acid sequences are shown in Fig. 4. Assuming that these alternative variants can be selected independently, then the extracellular region of *Pm*Dscam can potentially generate at least 54,756 different unique isoforms (26 × 81 × 26 = 54,756). We note that one of the Ig7 variants has an abnormal length (Fig. 4C), although the significance of this, if any, is unclear.

**Figure 5:**
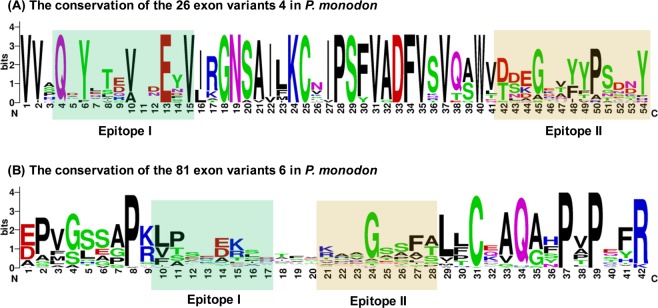
Differential sequence conservation of epitopes I and II of *Pm*Dscam. (**A**) Sequence logo representation of the conservation of exon 4 variants in *P. monodon*. (**B**) Sequence logo representation of the conservation of exon 6 variants in *P. monodon*. Bits on the y-axis indicate units of evolutionary conservation.

The first four Ig domains of Dscam are folded into a horse-shoe conformation, with parts of Ig2 and Ig3 contributing to two composite surface epitopes, epitope I and epitope II^21^. Although these two epitopes are not well conserved in insects^21^, they are highly conserved among crustaceans^15^. Epitope I is responsible for homophilic binding specificity, while it has been hypothesized that epitope II binds to non-Dscam ligands^21^. Here, we used PSIPRED (http://bioinf.cs.ucl.ac.uk/psipred) to locate the two epitopes in the Ig2 (exon 4) and Ig3 (exon 6) variants. Epitope I and epitope II sequence logos for exon 4 and exon 6 were then generated using WebLogo (http://wrblogo.berkeley.edu/). In exon 4, the sequence of approximately 12 amino acids before the conserved residue 16I, and the 13 amino acids after the conserved residue 41 V were identified as part of epitope I and II, respectively (Fig. 5A). In exon 6, the 8 amino acids after the conserved residue 9 K(R) completed epitope I, and the 8 amino acids before the conserved LLC motif completed epitope II (Fig. 5B).

### Expression of *Pm*Dscam isoform variants in hemocytes and nerve tissues

**Figure 6:**
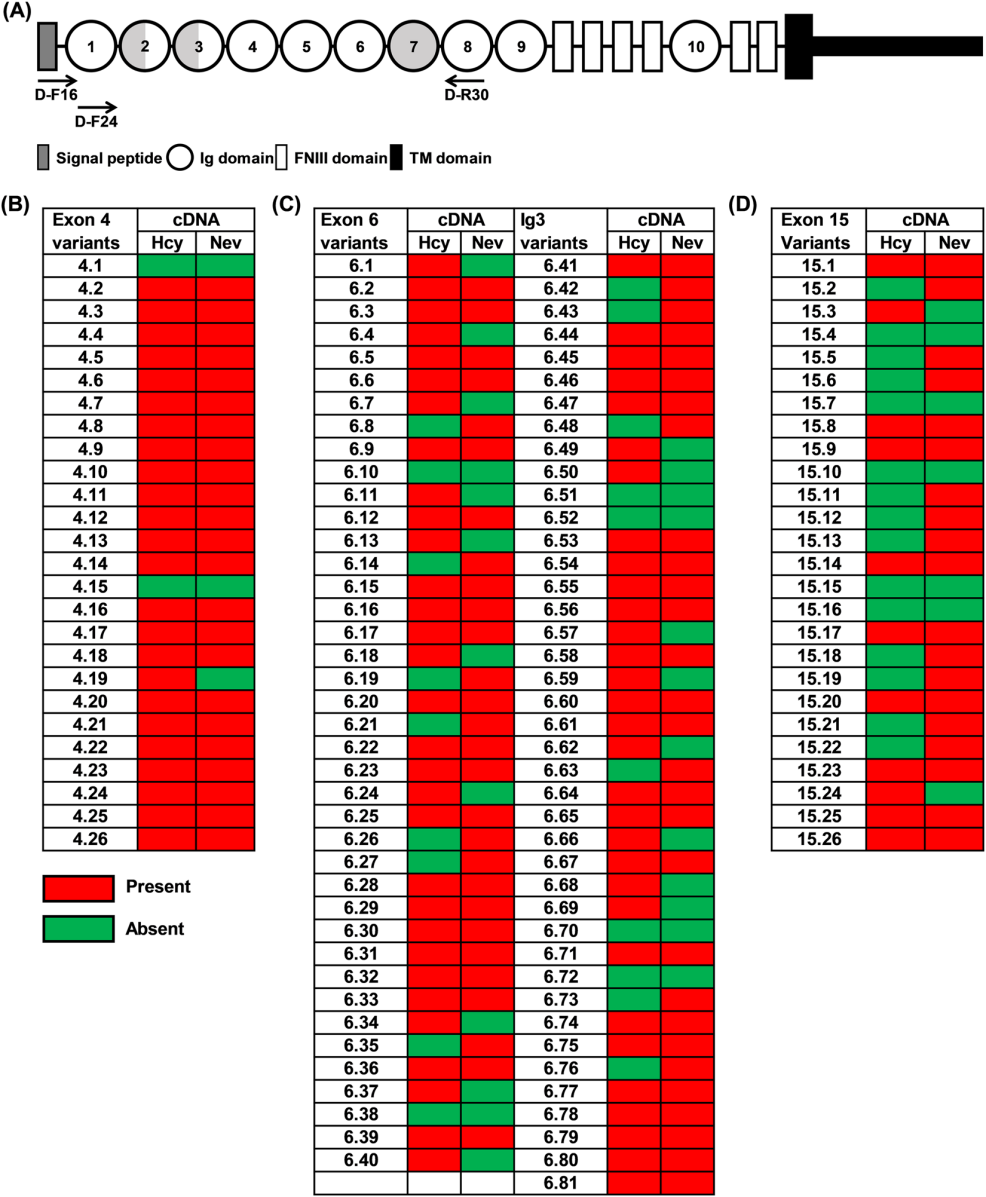
*Pm*Dscam isoform expression in hemocytes and nerve tissue. (**A**) Location of primers for PCR amplification and sequencing of *Pm*Dscam cDNA domain structure. (**B**) Exon 4 variants (**C**) exon 6 variants and (**D**) exon 15 variants detected in hemocyte (hcy) and nerve (nev) cDNA. Hemocyte and nerve samples were collected from 10 individual shrimp for total RNA extraction and cDNA synthesis. Twenty individual cDNA clones were obtained from each sample and their exon 4-exon 15 (Ig2-Ig7 domain) was sequenced. Red boxes represent detection of the isoform while green boxes represent non-detection of the isoform.

To check whether all of the isoform variants derived from the three hypervariable regions (exons 4, 6 and 15) are actually expressed in shrimp, and also to investigate whether there might be any differences in their expression patterns in immune-related cells (hemocytes) versus nerve tissue, amplicons spanning the hypervariable exons were amplified from hemocytes and nerve tissue from ten individual shrimp using gene specific primers (Fig. 6A). After cloning and sequencing, the obtained nucleotide sequences were BLASTed against our *Pm*Dscam gene database. As Fig. 6B–D shows, a small number of exon variants were not detected in either tissue. Among the exon 4 variants, isoform 1 and isoform 15 were not found in either hemocytes or nerve tissue (Fig. 6B). For exon 6, isoforms 10, 38, 51, 52, 70 and 72 were absent from both hemocytes and nerve (Fig. 6C), while isoforms 4, 7, 10, 15 and 16 of the exon 15 domain were also absent from both tissues (Fig. 6D). Curiously, we also note that the population distribution of the exon 15 isoforms was much more restricted in hemocytes than in nerve tissue (Fig. 6D). It remains unclear why these missing variants would fail to be expressed in one or both of these tissues.

### A complex cytoplasmic tail organization

**Figure 7:**
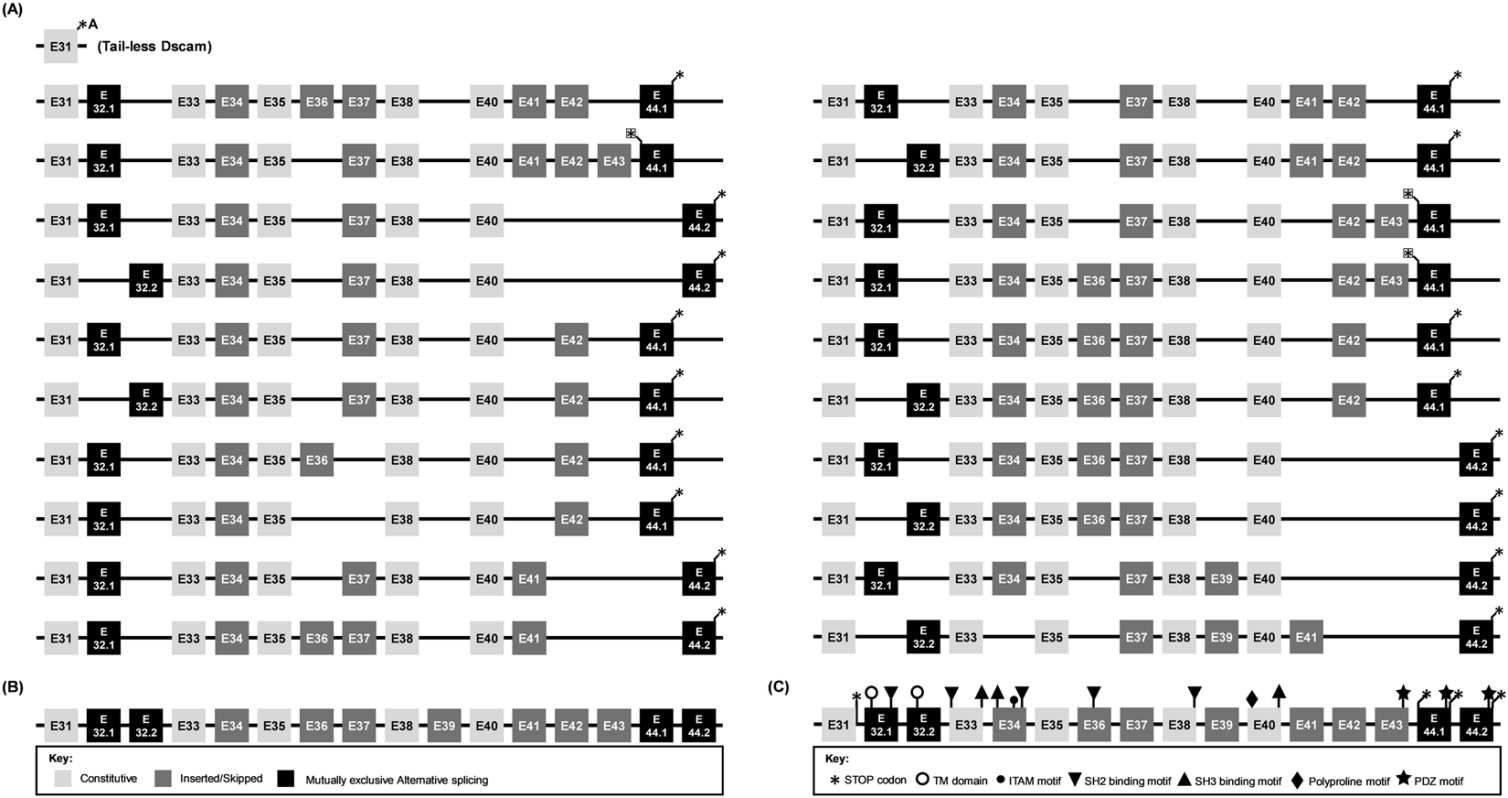
Organization of the *Pm*Dscam cytoplasmic tail. Exon numbers were determined according to the location of the exon in the *Pm*Dscam gene. (**A**) Schematic diagram showing the cytoplasmic tail exon combinations of 20 *Pm*Dscam contigs obtained from transcriptomics data. (**B**) Summary of exon types. Constitutive exons, inserted or skipped exons and alternative exon variants are shown as light grey boxes, dark grey boxes, and black boxes, respectively. (**C**) Cytoplasmic tail exons showing locations of common functional domains and motifs. Asterisks indicate a stop codon.

In our previous study^16^, although we successfully identified several cytoplasmic tail isoforms of *Pm*Dscam, we were only able to identify *Pm*Dscam element 0 to element 8 (with elements 0–5 corresponding to exons 31–38; the numbering of the elements corresponds to the exons in *Daphnia* Dscam). However, this earlier analysis contained several errors, and some of the downstream functional protein motifs were still missing. Here, using *P. monodon* Dscam protein sequences to search for additional putative exons against our transcriptomics database and then compared with *Drosophila* and *Daphnia*, we were able to identify the cytoplasmic tail of *Pm*Dscam from exon 31 to the stop codon in exon 44 (Fig. 7A). We named these exons according to the order in which they are located in the *Pm*Dscam gene. The amino acid sequences of each cytoplasmic exon are shown in Table 1. Differences between the naming system used in Chou *et al*.^16^ and the exons in Fig. 7 include: exons 36, 37 and 38, which were previously thought to be variants C, B and A of element 5, respectively, and the amino acid sequences from exon 39 to exon 44, which were grouped together as element 8. Two alternative kinds of transmembrane domain were found in exon 32; this is like *Drosophila*^8^ but unlike *Daphnia* Dscam^6^. Interestingly, mutually exclusive alternative splicing was also found in exon 44, with both of the two alternative exons containing the stop codon. In fact, the sequence for exon variant 44.2 is entirely contained within that of exon variant 44.1, and it is only because different reading frames are used to translate these two sequences that two distinct exons are expressed. Further, we found a special case that if exon 43 is included, it is always followed by exon variant 44.1, and the resulting nucleotide sequence will produce a stop codon in the very first amino acid of exon variant 44.1 (Fig. 7A). As noted previously^16^, in addition to the poly(A) tail that is located 364 nucleotides downstream of the 44.2 stop codon, there is also a stop codon and a poly(A) additional signal on the intron after exon 31 and before exon variant 32.1. When this intron is spliced and translation continues to the next exon (i.e. exon variant 32.1 or 32.2), the normal, membrane-bound form of Dscam is produced, but when this intron is included, it results in the production of the tail-less form of *Pm*Dscam. This tail-less form has been found in several crustaceans, but not in insects^10,16,24^. Bioinformatics analysis of exon organization in 20 *Pm*Dscam contigs containing the cytoplasmic tail found that exons 31, 33, 35, 38 and 40 are constitutively expressed, while exons 34, 36, 37, 39, 41, 42 and 43 can be either included or excluded (Fig. 7B). With the additional constraint that exon 43 is always followed by exon variant 44.1, this means that there must be at least 384 unique isoforms of the *Pm*Dscam cytoplasmic tail (i.e. 2^7^ × 3, where the presence or absence of exons 32, 34, 36, 37, 39, 41 and 42 account for the seven powers of 2, and the three valid combinations of exons 43 and 44 account for the multiplicative factor of 3).

**Table 1:** A comparison of the amino acid sequences of *Pm*Dscam cytoplasmic tail exons 31–44 with sequences of *D. melanogaster* exons 16–24 and *D. magna* exons 24–31.

Shrimp exon #	Tail element	Species	Amino acid sequence*	*D.melanogaster* exon # (Identity[%])	*D. magna* exon # (Identity[%])
31	E0	Pm, Lv	VAEYEVATLTLTG	16 (76.9)	24 (84.6)
32.1	E1A	Pm, Lv	GTIAPAREVPAFGAGDLPIYLNLNLIVPVVSAVVVIVLAIVIIC**YLRG**RNTPIK	17.1 (49.1)	25 (50)
32.2	E1B	Pm, Lv	ATLPPTVSDSRVTWLPDWWPKWLDLNVLVPVIATIVVIIVGIVVICVAVTRRKNGIENLR	17.2 (38)	—
33	E2	Pm, Lv	EEVYQQYQYNASMPPPSTMDKRHPGFREELG**YIPP**PNRKL***PPVP***GSQYNTCDRIKR	18 (64.3)	26 (55.4)
34	E3	Pm, Lv	GGGSGRGTHATWD***PRRP***MYEELSLHPPPGRRIPLGG***PPQP***LGSQDTLRS	19 (37.3)	27 (37.4)
35	E4	Pm, Lv	GGDDEICPYATFHLLGFREEMDPQQAGNNFQTFPHQNGHGSQQHFVNSPASRSM	20 (53.7)	28 (67.9)
36	E5	Pm, Lv	PRHGSGNYYSCVSGD**YTCG**HTPNEGHQ	20 (14.8)	29 (14.8)
37	E6	Pm, Lv	PRHGSGNYYSCVAGEYGPGG	20 (25)	—
38	E7	Pm, Lv	PPSSTYYSTVPGDMTASRMSNSTFSPT**YDDP**ARSDEESDQYGGSTYSGGGPYARAIDSVSQSGTAKRLS	21 (29)	29 (20.3)
39	E8	Pm, Lv	NGGHPPGAPVSGPQPSNHRFICK	—	29 (18.2)
40	E9	Pm, Lv	RGSTSGSAGQGSPEPLPLDSSGLGSSLNDSNNSTASNQFSEAECDHDLVQRNYG	22 (45.3)	29 (38)
41	E10	Pm	RHCAQTKP	23 (11.1)	30 (11.1)
42	E11	Pm, Lv	VKATKSTEEMRKLLDK	23 (53.3)	30 (60)
43	E12	Pm	KLNKT*	—	—
44.1	E13A	Pm, Lv	NEAAAHIQNGGLRMVSDEMNV*	24 (26.9)	31 (38.1)
44.2	E13B	Pm, Lv	EMKQLPTFKMEA*	—	—

*Underlining: transmembrane domain (TM); bold: SH2 binding site; bolded italics: SH3 binding site; box: polyproline motif; italics: PDZ motif; and asterisk: stop codon.

The transmembrane domain (TM) is located in either exon variant 32.1 or exon variant 32.2 (Fig. 7C; Table 1. The other functional motifs of Dscam, which are highly conserved among crustaceans and insects, were predicted with the simple modular architecture research tool (SMART) version 4.0 and are also shown in Fig. 7C and Table 1. Putative Scr homology 2 (SH2) binding motifs were predicted in exon variant 32.1, and exons 33, 34, 36 and 38, while putative Scr homology 3 (SH3) binding motifs were predicted in exons 33, 34 and 40. An immunoreceptor tyrosine-based activation motif, ITAM (consensus: YXXL), was predicted in exon 34. A polyproline motif was predicted in exon 40, and Zo-I protein (PDZ) domain motifs were predicted in exon 43 and exon variants 44.1 and 44.2. However, we were unable to identify an immunoreceptor tyrosine-based inhibitory motif (ITIM) in any of the *Pm*Dscam exon variants.

Table 1 also shows the result of aligning the amino acid sequence of the *Pm*Dscam cytoplasmic tail against the cytoplasmic tail domains of both *Drosophila melanogaster* (AF260530) and *Daphnia magna* (ACC65887). *Pm*Dscam exons 31–44 correspond to exons 16–24 of *D. melanogaster* Dscam and exons 24–31 of *D. magna* Dscam. *Pm*Dscam exon 39 can be found in *D. magna* but not in *D. melanogaster*, while *Pm*Dscam exon variant 32.2 and exon 37 are absent from *D. magna*. In contrast to other crustacean and insect Dscams, we note that exon 43 and exon variants 44.2 have so far been found only in shrimp. We further note that exons 41 and 43 were found in *P. monodon* and not in *L. vannamei* Dscam. Finally, the *Pm*Dscam cytoplasmic tail includes important protein motifs that correspond to those in *Drosophila* and *Daphnia* Dscam, even though many of the amino acid sequences in each exon share a percent identity of less than 50% (Table 1).

The entire annotated *Pm*Dscam gene has now been uploaded to NCBI (NCBI accession number: MK838771).

### The *Pm*Dscam ORF

**Figure 8:**
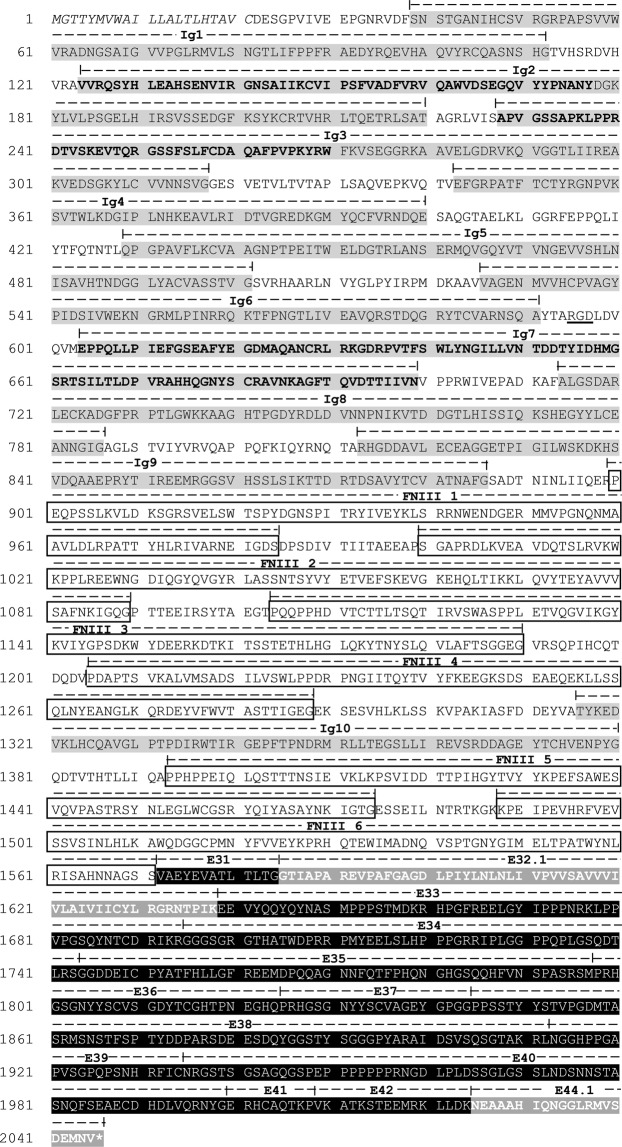
The amino acid sequence of a *Pm*Dscam isoform. In the extracellular region, putative signal peptides are in italics, and Ig domains are shaded light grey. The variable sequences in Ig2, Ig3 and Ig7 are in bold. FNIII domains are boxed. A conserved RGD motif is indicated by underlining. In the cytoplasmic tail, which is rendered in reverse contrast, the constitutive and optional domains are shaded black, while the mutually exclusive alternatively spliced domains are in bold against a light grey background. The asterisk indicates the stop codon.

An example of the complete full-length *Pm*Dscam, including both the extracellular region and the cytoplasmic tail, is shown in Fig. 8. The open reading frame (ORF) of this *Pm*Dscam isoform contains 6,135 bp encoding a predicted protein of 2,045 amino acid residues, although the lengths of the nucleotide and amino acid sequences of other isoforms will vary as a result of alternative splicing and skipped exons. The putative signal peptide predicted by Signal P3.0 domain analysis is located at the N-terminus. Domain homology analysis using SMART software showed that the deduced amino acid sequence contained ten tandem repeat immunoglobulin domains (Ig), six fibronectin type III domains (FNIII) and up to thirteen exons in the cytoplasmic tail. The hypervariable sequences in Ig2, Ig3 and Ig7 are indicated. The conserved cell attachment RGD motif (Arg-Gly-Asp) is located between the Ig6 and Ig7 domains at amino acids 595 to 597. The two exons in the cytoplasmic tail with mutually exclusive alternative splicing (i.e. exons 32 and 44) are also indicated.

## Discussion

During the past decade, several approaches, including BAC end sequencing, linkage map construction, transcriptome sequencing and whole-genome sequencing, have been used to investigate the genome and genetic properties of crustaceans^26,27^. However, the long and highly repetitive sequences of the crustacean genome cause difficulty in genome assembly and other genetic studies^26,28^. Furthermore, crustacean genomes show substantial variations in size. For example, the genomes of caridean shrimp (*Exopalaemon carinicauda*) and white shrimp (*Litopenaeus vannamei*) are 5.73 and 2.3 Gb, respectively^28,29^, while the *Penaeus monodon* genome size was estimated to be ~2.1 Gb. In the present study, the *P. monodon* whole-genome sequence was assembled using state-of-the-art genomics techniques, including a combination of short read Illumina and long read PacBio sequencing and hybrid assembly. From this whole genome sequence, we obtained a *Penaeus monodon* Dscam (*Pm*Dscam) gene of ~266 kb that was subjected to correction and analysis (Fig. 2A).

We reported previously^16^ that *Pm*Dscam has a typical Dscam domain architecture similar to arthropod Dscam^9^. The extracellular region has 10 immunoglobulin domains and six fibronectin III domains, i.e., [Ig1-Ig9]-[FNIII 1-FNIII 4]-[Ig10]-[FNIII 5-FNIII 6], with half of the second and third Ig domains and the entire Ig7 domain encoded by arrays of near-duplicate exons. The FNIII6 of the extracellular region is followed by a transmembrane domain and a cytoplasmic tail^5,6^. The diversity of the hypervariable regions, i.e. the Ig2, Ig3 and Ig7 domains, arises from mutually exclusive alternative splicing, which ensures that in mature mRNA there is only one exon variant selected from each array cluster^7^. In the present study, we found that the *Pm*Dscam gene has a total of 44 exons, including three hypervariable regions in the extracellular region, i.e. the extracellular exon variant clusters 4, 6, 15, and two cytoplasmic tail variant exon clusters (32 and 44), each of which consists of two mutually exclusive alternatively spliced variants (Fig. 3A,B). In contrast to our previous study, which reported finding 28, 43 and 19 alternative sequences for N-terminal Ig2, N-terminal Ig3 and the entirety of Ig7, respectively^16^, Fig. 4 shows that the correct numbers are in fact 26, 81 and 26. There are two reasons for these discrepancies. In the previous study, isoforms with only a single amino acid difference were counted as distinct isoforms even though they were more likely to have resulted from sequencing errors. This would have artificially inflated the earlier figure. Conversely, a number of isoforms were simply not found in the Chou *et al*.^16^ study. The new sequencing methods used here have now corrected both of these errors.

Our present results also show that, compared to the three hypervariable regions in other arthropods, *Pm*Dscam has the highest number of total possible combinations^3,8,15,22^. That is, as noted above, since there are 54,756 possible combinations that can be generated by the extracellular region, and 384 more that can be produced by the cytoplasmic tail, *Pm*Dscam can express 54,756 tailless isoforms plus 21,026,304 isoforms (i.e. 54,756 × 384) that are membrane-bound. Against this total of 21,081,060 isoforms, by comparison, there are only 30,600 Dscam isoforms in crab, 19,008 in *Drosophila* and 3,264 in *Daphnia*^6,8,15^.

The presence of Dscam in both nerve cells and immune-related cells such as hemocytes implies it might have a role in both the nervous and immune systems^2,4,30^. Assuming that these two roles are functionally distinct, we might further expect to see different populations of Dscam isoforms in these two tissues. However, when we compared the expression of the *Pm*Dscam hypervariable exons in hemocytes and nerves, we found that the expressed variants for exon 4 were very similar (Fig. 6B). Curiously, we also found that there was a higher level of amino acid similarity between the exon 4 variants than between the variants of the other two hypervariable exons (Fig. 4). For exon 6, different isoforms were expressed even though the overall population diversity was similar (Fig. 6C). Lastly, we observed a high diversity of exon 15 variants in nerve tissues compared to hemocytes (Fig. 6D). Overall, *P. monodon* Dscam populations are therefore unlike those of *Drosophila* and *Daphnia*, both of which show less diversity in all three of the corresponding exons in their immune cells compared to their nervous systems^2,6^.

The protein structure of Dscam’s the extracellular domain supports its involvement in binding interactions. Parts of the Ig2 and Ig3 domains form a horseshoe configuration which allows independent interactions on either side of the horseshoe^21^. Surface epitope I is important for homophilic binding specificity and is made up to N-terminal sequences from exon 4 and exon 6, while epitope II, which may be involved in non-Dscam binding, is made up of C-terminal sequences from the same two exons. In *Pm*Dscam, the two epitopes (Fig. 5) presumably fulfill the same functions. However, we also note that the amino acid sequences of the *Pm*Dscam epitopes have a high similarity to those of *Es*Dscam^15^, suggesting that, as in crab, *Pm*Dscam may bind with specific pathogens and regulate phagocytosis.

Sequences derived from our transcriptomics data were used to determine the location of the unknown exons in the cytoplasmic tail of shrimp Dscam. Unlike Dscam from other arthropods, *Pm*Dscam not only has two alternative variants that encode for the transmembrane domain, but also two alternative variants that encode for the stop codon in the cytoplasmic tail (Fig. 7B). *Pm*Dscam also includes instances of several other functional domains that are conserved in arthropod Dscams, including the SH2-binding motif, the SH3-binding motif, the ITAM motif, the polyproline motif and the PDZ motif (Fig. 7C; Table 1). These small binding motifs are involved in specific protein-protein interactions in cellular signal transduction^31,32^. For example, the SH2/SH3-binding motif interacts with Dock to activate axon guidance in *Drosophila*^5^, while the ITAM motif is involved in downstream protein tyrosine kinase (PTK)-mediated immunoreceptor signaling after ligand binding and it regulates the expression of surface membrane receptors^6,33^. The PDZ motif determines which exons are present on the cytoplasmic tail^34^. Interestingly, no immune tyrosine-based inhibition motif (ITIM) (I/S/V/LXYXXV/L) was found in *Pm*Dscam. The ITIM motif is also missing from crab Dscam^23,35^, and implies that these two crustaceans may have only positive transmembrane signaling. In *Daphnia*, the cytoplasmic tail can include or exclude the ITIM or ITAM motif, implying variable signal capacity^6^. Like other arthropod Dscams, *Pm*Dscam contains an RGD (Arg-Gly-Asp) motif that is recognized by integrin family members^36^. In *Pm*Dscam, this is located between Ig6 and Ig7 in the extracellular region. As also seen in other arthropod Dscams^37–39^, alternative splicing produces variable exons in the cytoplasmic tail (Fig. 7B). Depending upon the RNA splicing, exons in the cytoplasmic tail can be either excluded or included, which can affect both the length and the frame shift of *Pm*Dscam’s reading frame. Similarly, in *Daphnia*, if exon 30 was excluded, the reading frame for exon 31 was shifted, whereas exclusion of exon 27 did not affect the reading frame^6^. However, while inclusion or exclusion of exons in *Daphnia* Dscam can result in the absence of an ITIM motif and PDZ domain^6^, splicing of *Pm*Dscam cytoplasmic tail exons results only in the absence of the ITAM motif and not the PDZ domain (Fig. 7C). In *Pm*Dscam, there is a PDZ domain in the C-terminal regions of exon 43, and the exon variants 44.1 and 44.2 (Fig. 6C, Table 1), suggesting that these mutually exclusive alternative PDZ domains might interact with different proteins located in various parts of the cellular membrane^39^. Isoforms with or without these motifs may have important differences in signaling capacity and in their ability to regulate the expression of surface membrane receptors^40^.

## Conclusions

Combining all the data obtained from genomics, transcriptomics and cDNA, we successfully generated an in-house database (http://pmdscam.dbbs.ncku.edu.tw/) of *Pm*Dscam which was sufficient to support BLAST function ability for nucleotide and amino acids sequences of the extracellular regions and cytoplasmic tail. This database should be useful for researchers who need to identify which of the hypervariable exons were used to produce a particular isoform. The sequence of this *Pm*Dscam gene as well as our in-house database should be useful resources for future research.

## Methods

### Whole-genome sequencing

To construct the complete Dscam gene (*Pm*Dscam) for the tiger shrimp *Penaeus monodon*, we first used a combination of traditional, next-generation, and new third-generation sequencing strategies to assemble a polished draft of the entire *P. monodon* genome (Fig. 1A). For the Illumina whole-genome sequencing, the standard phenol–chloroform procedure was used to extract genomic DNA from the muscle tissue of an adult female (F09) collected from the coastal waters of Taiwan. Using the standard operating protocol provided by Illumina (San Diego, CA, USA), two different types of insert library for sequencing were constructed: paired-end libraries for small inserts (180, 350, and 500 bp), and mate-pair libraries for large inserts (2, 5, and 8 kb) (Table S2). Paired-end sequencing was performed using the Illumina HiSeq platform, and a total of 585.60 Gb of raw reads (293.03 Gb from the small insert libraries and 292.57 Gb from the large insert libraries) were generated (Table S2). After quality control removing low-quality reads as well as PCR-replicates and adapter sequences, we obtained 486.22 Gb (224.06X of genome coverage) of clean data for subsequent assembling.

In addition, to improve the assembly quality and increase the scaffold N50, we adopted PacBio (Pacific Biosciences) single-molecule real-time sequencing strategy. Pleopod genomic DNA (F40) was extracted using the Blood and Cell Culture DNA Midi Kit (Qiagen) for construction of a 20-kb insert-size library. A total of 29 SMRTcells were sequenced on the PacBio RS II platform, producing ~17.9 Gb of long reads data with a read length N50 of 11.6 kb (mean 9.14 kb) (Table S2).

### *De novo* genome assembly

As Fig. 1A shows, for the preliminary genome assembly, we first assembled the Illumina short reads using two different programs, *Allpaths-LG*^41^ and *Velvet*^42^, separately. The ALLPATHS assembly had a higher N50 length (6,606 bp vs. 2,458 bp) and a much lower contig number (251,428 vs. 2,003,807) than the VELVET assembly, but the total contig length (1,101,722,092 bp) was only half of the VELVET assembly (2,167,365,623 bp). The VELVET assembly contig length was very close to the full length of the *P. monodon* genome (~2.17 Gb) as estimated by flow cytometry^43^.

To improve the scaffold N50, a third assembly was produced. This was a hybrid assembly combining both the Illumina short reads and PacBio long reads data. However, due to computational limitations, not all Illumina data were used for this assembly. Following Chakraborty *et al*.^46^, we first assembled approximately 140 Gb of Illumina data (obtained from the 180 bp insert library) using *Platanus*^44^; this assembly was then combined with all the PacBio long reads using *DBG2OLC*^45^ to produce the hybrid assembly.

To obtain an optimum assembly that had both contiguity and completeness and could serve as a practical genome database, the three assemblies were sequentially merged using *quickmerge*^46^. For this process, the DBG2OLC assembly (most contiguous and least complete) was merged to the ALLPATHS assembly (the next most contiguous but more complete), and the result was then merged to the VELVET assembly to produce the first draft M2 assembly (Fig. 1A; Table S1). Default merging parameters (python merge_wrapper.py ${hybridpath} ${selfpath} -hco 5 -c 1.5 -l 10000) were used, with the exception of the −1 parameter (minimum size cutoff for seed contigs for merging) due to the low average contig size across the genome, which would have prevented merging had the ordinary cutoff been used. The M2 assembly was polished using one round of *Quiver*^47^ error correction and one round of *Pilon*^48^ error correction, again as described in Chakraborty *et al*.^46^. All available PacBio data and all available non-matepair Illumina data were used for polishing. The polished M2 assembly of the *P. monodon* genome was then used to produce a draft sequence of the *P. monodon* Dscam gene.

Next, in order to fill the gaps which were still found in some parts of the *Pm*Dscam gene (please see Fig. 2A) and to confirm the sequences, Sanger sequencing was performed using cDNA and genomic DNA samples. Total RNA samples were isolated from hemocytes using REzolTM C&T reagent (Protech Technology, Taiwan) according to the manufacturer’s protocol. First-strand cDNA synthesis was performed using SuperScript® ll Reverse Transcriptase (Invitrogen) according to the manufacturer’s instructions. Genomic DNA was extracted from the pleopods of individual shrimp using a DNA extraction kit (GeneReach Biotechnology Corp.). The hemocyte cDNA and pleopod genomic DNA were used as templates for PCR amplification of the exon and intron fragments using gene specific primers (Table 2). The PCR products were separated by agarose gel electrophoresis and purified prior to cloning. The purified DNA fragments were cloned into RBC T&A cloning vector (RBC Bioscience, Taiwan) and sequenced using M13F and M13R universal primers.

**Table 2:** Nucleotide sequences of the primers used.

Primer	Primer sequence (5′—3′)
D-F16	5′ ATGGGCACTACCTATATG 3′
D-F24	5′ CTGATCTTCCCTCCCTTC 3′
D-R30	5′ CAAGATCGCGATAGTCAC 3′
**Introns/Exons confirmation**	
D-Ig4-F	5′ TCGAGACTGTGCTCACTGT 3′
D-Ig4-R	5′ GTGTCAATGCGAAGAACAGC 3′
D-Ig6-F	5′ TGCAGTTCACACAAATGATGGA 3′
D-Ig6-R	5′ AACAATGAGGGTGCCATTG 3′
D-Ig8-F	5′ CACGCTGGATTGTGGAAC 3′
D-Ig9-R	5′ TTCCAAAAGCATTGGTAGCC 3′
D-gFNIII1-F	5′ AGAACATGGCAGCTGTCTTG 3′
D-gFNIII3-R	5′ TCACCACCTGATGTGAAAGC 3′
D-gFNIII6-F	5′ GAAGCCTGAGATTCCTGAAG 3′
D-gE0-R	5′ TCCAGTGAGAGTCAATGTAG 3′
D-EL1(A)-F	5′ TCTCTGCTGTAGTCGTCATC 3′
D-EL1(B)-R	5′ CTCAACATCGCAGTCTAAAG 3′
D-I-E1B-F	5′ CCCAGTTATTGCCACTATCG 3′
D-I-E2-R	5′ TAGCAAGTCTCCCTGGAATC 3′
D-I-E4-F	5′ TCTGCCCTTATGCTACCTTC 3′
D-I-E5-R	5′ GGGTATGGCCACAAGTATAG 3′
D-gE8-F	5′ CCTCAACCCAGCAACCATAG 3′
D-gE9-R	5′ CTCCATAATTACGCTGCACAAG 3′
D-gE9-F	5′ AACCGTGGAAGCACCTCT 3′
D-gE10-R	5′ TTGGTTTGTGCACAATGTCT 3′
D-gE11-F	5′ CCAAGAGCACAGAGGAGATG 3′
D-gE12A-R	5′ GGTCTTATTCAGTTTCCTCG 3′

### Transcriptome sequencing and assembly

For the transcriptome sequencing, *Penaeus monodon* postlarvae were challenged with Nidovirus. Pooled stomach samples were taken from the postlarvae in both the control and Nidovirus-infected group at 48 h post infection. A RNeasy Mini Kit (Qiagen) was used to extract the total RNA following the manufacturer’s instructions. Quantification and quality control of the RNA samples were determined by an RNA 6000 Nano kit with an Agilent2100 Bioanalyzer (Agilent Technologies Inc.). Paired‐end sequencing was performed on an Illumina NextSeq500 (Genomics BioSci & TechCo.), and the paired‐end reads were assembled using Trinity (v.2.1.1^49^) with strand‐specific mode (SS_lib_type RF). For functional classification, annotations were determined using BLAST with the Flybase database, and analysis was conducted using PANTHER^50^. The gene annotations were determined using BLAST with the NCBI‐PM and EMBL‐CDS databases, and analysis was conducted using the ContigViews^51^ web server.

The transcriptomics database was used to search for the remaining exons located in the cytoplasmic tail region. To obtain the sequence of the cytoplasmic tail, several conserved sequences of *Pm*Dscam (Table S3)^16^ were first used to search against the transcriptomics database. Then, all of the nucleotides were translated to amino acid sequences, and BLASTed against the NCBI database. The obtained sequences were analyzed and identified as both nucleotide and amino acid sequences in each exon. Finally, the *Pm*Dscam gene sequence was searched for the nucleotide sequences of each exon to find the location of those exons on the *Pm*Dscam gene (Fig. 1B). All of the exon sequences for *Pm*Dscam have been uploaded to our in-house database.

### Identification of *Pm*Dscam hypervariable regions and sequence analysis

To obtain the hypervariable sequences of the *Pm*Ds*cam* exons in Ig2, Ig3 and Ig7, we first searched the corrected M2 assembly to find the locations of the conserved amino acid sequences of previous known *Pm*Dscam isoform variants from each domain^16^. To ensure that every potential isoform variant was included, we then aligned all matching variants and used the conserved sequences from each variable region as a guide to search for all the possible exons in the *Pm*Dscam gene sequences. Like the other *Pm*Dscam exons, the hypervariable region exons (i.e. exon 4, 6 and 15) were named according to their order of the location in the *Pm*Dscam gene.

### Expression of *Pm*Dscam isoform variants in hemocytes and nerve tissues

To investigate the expression of the *Pm*Dscam hypervariable exons, hemocytes and nerve tissues were collected from ten individual shrimp. For the hemocyte samples, hemolymph was drawn from the ventral sinus using a sterile 1-ml syringe with anticoagulant solution and centrifuged at 10,000 g for 1 min at 4 °C to separate the hemocytes. Then, for both the hemocytes and excised nerve tissue, total RNA was extracted from each sample using REzol^TM^ C&T reagent (Protech Technology, Taiwan) following the manufacturer’s instructions. The extracted mRNA was used as a template to synthesize first-strand cDNA with SuperScript® ll Reverse Transcriptase (Invitrogen) according to the manufacturer’s instructions. To obtain the cDNA sequence of the Ig2, Ig3 and Ig7 variable exons, we performed the polymerase chain reaction (PCR) using 2 nested sets of oligonucleotide primer pairs specific to *Pm*Dscam. The first amplification used the primers D-F16 and D-R30 (Table 2). The PCR reaction mixture contained 0.2 mM dNTP, 1.5 mM MgCl2, 0.2 µM of each primer and 2X Taq DNA Polymerase Mastermix-RED (Bioman). The PCR reaction was carried out as follows: 94 °C for 5 min, then 35 cycles of 94 °C for 30 sec, 55 °C for 30 sec, 72 °C for 2 min, followed by a final extension at 72 °C for 10 min. The PCR product was then diluted and used as the template for the second amplification of the nested PCR with the primers D-F24 and D-R30 (Table 2) in the presence of 1 unit of Takara Ex taq polymerase (Takara). The PCR reaction was carried out as described above. The PCR products were purified and cloned into RBC T&A cloning vector (RBC Bioscience, Taiwan). Individual colonies (n = 20) containing insert fragments from each sample were selected randomly and sequenced using M13F and M13R universal primers. BLAST was used to check that the obtained sequences corresponded to our *Pm*Dscam gene database. Isoform sequences were aligned with Crustal Omega (http://www.ebi.ac.uk/uniprot/).

### The *Pm*Dscam database

The *Pm*Dscam database was constructed on a LAMP (Linux + Apache + MySQL + PHP) system. The web interface is written in PHP. BLAST algorithms^52^, including blastn, blastp and blastx, were used for sequence alignment, with the e-value set to 10e-10 as default. There are a total of 175 *P. monodon* Dscam exons and/or exon variants in the *Pm*Dscam database. Users can input multiple sequences in FASTA format to perform an analysis. All the blast results for each sequence will be shown.

## Supplementary information

Supplementary info
