# Supplementary material for: The gene structure and hypervariability of the complete Penaeus monodon Dscam gene
Source: Sci Rep. 2019 Nov 12;9:16595. doi: 10.1038/s41598-019-52656-x (PMC6851185; doi:10.1038/s41598-019-52656-x)
Supplement: Supplementary file 1 — Supplementary info [file 41598_2019_52656_MOESM1_ESM.doc]

**Supplemental Information**

**----------------------------------------------------------------------------------------------------**

Running title:

**The gene structure and hypervariability of the complete *Penaeus monodon* Dscam gene**

Kantamas Apitanyasai^1,2^, Shiao-Wei Huang^3^, Tze Hann Ng^1,2^, Shu-Ting He^1,2^, Yu-Hsun Huang^1,2^, Shen-Po Chiu^1^, Kuan-Chien Tseng^4^, Shih-Shun Lin^5^, Wen-Chi Chang^6^, James G. Baldwin-Brown^7^, Anthony D. Long^7^, Chu-Fang Lo^1,2^, Hon-Tsen Yu^3^, Han-Ching Wang^1,2*^

**^1^**Department of Biotechnology and Bioindustry Sciences, National Cheng Kung University, Tainan, Taiwan

**^2^**International Center for the Scientific Development of Shrimp Aquaculture, National Cheng Kung University, Tainan, Taiwan

**^3^**Department of Life Sciences, National Taiwan University, Taipei, Taiwan

^4^Department of Life Sciences, National Cheng Kung University, Tainan, Taiwan

^5^Institute of Biotechnology, National Taiwan University, Taipei, Taiwan

^6^Institute of Tropical Plant Sciences, National Cheng Kung University, Tainan, Taiwan

^7^Department of Ecology and Evolutionary Biology, University of California, Irvine, Irvine, California, USA

**Corresponding author:**

Han-Ching Wang, Professor

E-mail: wanghc@mail.ncku.edu.tw

Phone: +886-6-2757575 ext 58219

Address: Department of Biotechnology and Bioindustry Sciences, National Cheng Kung University, Tainan, Taiwan

**Table S1**. Summary of the four genome assemblies

| **Assembly** | **Data and programs used** | **Genome size**  **assembled** | **#Total contigs**  **(*10^3^)** | **Contig length**  **N50** |
| --- | --- | --- | --- | --- |
| (1) ALLPATHS | All Illumina short reads (486.222 Gb) was assembled by *Allpaths-LG* | 1.101 Gb | 251.4 | 6.6 kb |
| (2) VELVET | All Illumina short reads (486.222 Gb) was assembled by *Velvet* | 2.167 Gb | 2003.8 | 2.4 kb |
| (3) DBG2OLC | ~140 Gb of Illumina short reads was firstly assembled by *Platanus*, then combined with 17.393 Gb of PacBio long reads by *DBG2OLC* | 0.767 Gb | 28.6 | 30.5 kb |
| (4) M2 | The DBG2OLC assembly were merged to the ALLPATHS assembly by *Quickmerge*; this was then merged to the VELVET assembly | 2.600 Gb | 2200.0 | 5.1 kb |

**Table S2**. Summary of the whole-genome sequencing data.

| **Platform** | **Library type** | **Insert size** | **NumberReads** | **ReadLength** | **NumberReads after trim** | **Avg ReadLength after trim** | **TotalBase**  **after QT (bp)** | **Coverage (X)** | **Note** |
| --- | --- | --- | --- | --- | --- | --- | --- | --- | --- |
| Illumina | Paired-end | 180 bp | 1,696,204,966 | 100 bp | 1,656,882,369 | 94.8 bp | 157,145,433,519 | 72.4 | R1+R2 |
|  |  | 350 bp | 643,018,862 | 100 bp | 619,848,502 | 94.3 bp | 58,474,824,576 | 26.9 | R1+R2 |
|  |  | 500 bp | 562,068,632 | 100 bp | 486,012,079 | 89.2 bp | 43,354,635,668 | 20.0 | R1+R2 |
|  | Mate-pair | 2 kb | 980,700,602 | 100 bp | 880,209,591 | 84.7 bp | 74,530,767,232 | 34.3 | R1+R2 |
|  |  | 2 kb | 104,982,017 | 210 bp | 100,439,244 | 186.4 bp | 18,720,711,212 | 8.6 | R1 |
|  |  | 2 kb | 104,982,017 | 125 bp | 100,439,244 | 110.6 bp | 11,107,884,104 | 5.1 | R2 |
|  |  | 2 kb | 54,244,124 | 210 bp | 51,983,147 | 164.1 bp | 8,527,895,825 | 3.9 | R1 |
|  |  | 2 kb | 54,244,124 | 175 bp | 51,983,147 | 141.8 bp | 7,372,829,503 | 3.4 | R2 |
|  |  | 5 kb | 818,372,514 | 100 bp | 767,728,651 | 86.7 bp | 66,569,692,722 | 30.7 | R1+R2 |
|  |  | 5 kb | 84,545,083 | 210 bp | 76,022,861 | 179.5 bp | 13,644,892,067 | 6.3 | R1 |
|  |  | 5 kb | 84,545,083 | 125 bp | 76,022,861 | 105.3 bp | 8,005,735,136 | 3.7 | R2 |
|  |  | 8 kb | 77,156,219 | 210 bp | 71,311,590 | 169.2 bp | 12,068,791,883 | 5.6 | R1 |
|  |  | 8 kb | 77,156,219 | 125 bp | 71,311,590 | 93.9 bp | 6,698,655,233 | 3.1 | R2 |
| **Illumina total** | |  |  |  |  |  | **486,222,748,680** | **224.06** |  |
| **PacBio** | | 20 kb |  |  | 1,903,249 | (mean) 9.14 kb | **17,393,019,345** | **8.02** |  |
| **Illumina + PacBio total** | |  |  |  |  |  | **503,615,768,025** | **232.08** |  |

*P. monodon* genome size (2.17 Gb) was estimated by flow cytometry (Huang *et al*.^43^)

**Table S3.** Nucleotide sequences for blast against transcriptome database

| **Domain region** | **Nucleotide sequences** |
| --- | --- |
| **Extracellular region** |  |
| **Ig1** | AGCAACTCAACTGGCGCCAATATCCACTGCTCTGTTCGAGGACGACCCGCCCCCTCCGTCGTTTGGGTTCGCGCTGACAATGGCTCCGCTATCGGTGTTGTT  CCTGGCCTTAGGATGGTTCTCTCCAACGGCACCCTGATCTTCCCCCCCTTCCGCGCCGAGGACTACCGTCAGGAAGTGCACGCCCAGGTCTACCGCTGCCA  GGCCTCCAACTCCCACGGC |
| **Ig2 (C-terminal)** | GACGGCAAGTACTTGGTCCTTCCCTCCGGCGAACTACACATCCGCTCCGTCAGCTCCGAAGACGGTTTCAAGAGCTACAAGTGCCGCACCGTGCATCGCCT  CACCCAGGAAACACGCCTCTCCGCCACCGCTGGACGTCTTGTGATCTCC |
| **Ig6** | GTTGCTGGAGAAAACATGGTTGTACACTGCCCCGTTGCTGGATATCCCATTGACTCTATTGTTTGGGAAAAGAATGGTCGCATGCTGCCCATCAACCGCCGC  CAGAAGACATTCCCCAATGGCACCCTCATTGTTGAAGCTGTCCAACGCTCCACTGACCAAGGAAGATACACCTGTGTTGCCCGTAACAGCCAGGCT |
| **Cytoplasmic tail** |  |
| **Exon 31** | GTTGCTGAATATGAAGTTGCTACATTGACTCTCACTGGA |
| **Exon 32.1** | GGTACTATTGCCCCGGCTCGTGAAGTACCCGCCTTCGGGGCTGGAGATCTCCCGATCTACCTCAACCTAAACCTCATCGTTCCTGTGGTCTCTGCTGTAGTC  GTCATCGTTTTGGCCATCGTTATCATCTGCTATCTGAGAGGTCGCAATACTCCAATCAAA |
| **Exon 32.2** | GCCACGCTGCCGCCAACTGTTTCGGACAGCCGAGTAACCTGGCTTCCTGATTGGTGGCCGAAGTGGCTGGATCTGAATGTCCTGGTCCCAGTTATTGCCAC  TATCGTCGTCATCATTGTGGGCATTGTTGTCATCTGTGTTGCAGTAACTCGTCGCAAGAACGGCATTGAGAACTTGAGACTGCGA |
| **Exon 35** | GGTGGTGATGATGAGATCTGCCCTTATGCTACCTTCCACCTTCTGGGCTTCCGTGAGGAAATGGACCCCCAACAAGCTGGCAACAACTTCCAGACTTTCCC  CCACCAGAATGGCCATGGCTCACAACAACACTTCGTCAACTCCCCTGCCTCAAGGAGCATG |
| **Exon 38** | CCTCCAAGCAGCACCTACTACTCTACTGTCCCTGGCGACATGACCGCTTCTCGCATGAGCAACTCAACTTTCTCTCCGACCTACGATGACCCTGCCCGCTCT  GATGAAGAGAGTGACCAATATGGTGGATCTACTTACTCTGGTGGTGGACCCTATGCTCGAGCCATTGACTCTGTGTCACAGTCTGGCACTGCTAAACGCCTT  ACTAAC |

**Figure S1.** Cumulative contig length for four genome assemblies, i.e., ALLPATHS, VELVET, DBG2OLC, and M2 assemblies. Merged assembly M2 were larger than the largest unmerged assemblies (VELVET assembly). One explanation for this phenomenon is that each assembly contained unique content. Another explanation is that small contigs cannot be effectively mapped to each other; therefore, matching contigs from both assemblies in a merge were retained, adding redundancy to the merged assembly.
